# Ecological Adaptation of Wild Peach Palm, Its *In Situ* Conservation and Deforestation-Mediated Extinction in Southern Brazilian Amazonia

**DOI:** 10.1371/journal.pone.0004564

**Published:** 2009-02-24

**Authors:** Charles R. Clement, Ronaldo P. Santos, Sylvain J. M. Desmouliere, Evandro J. L. Ferreira, João Tomé Farias Neto

**Affiliations:** 1 Instituto Nacional de Pesquisas da Amazônia, Manaus, Amazonas, Brasil; 2 Instituto Nacional de Colonização e Reforma Agrária, Manaus, Amazonas, Brasil; 3 Instituto Nacional de Pesquisas da Amazônia, Nucleus – Acre, Rio Branco, Acre, Brasil; 4 Embrapa Amazônia Oriental, Belém, Pará, Brasil; Stanford University, United States of America

## Abstract

**Background:**

The Arc of Fire across southern Amazonia seasonally attracts worldwide attention as forests are cut and burned for agricultural expansion. These forests contain numerous wild relatives of native South American crops, such as peach palm.

**Methodology/Principal Findings:**

Our prospecting expeditions examined critical areas for wild peach palm in the Arc of Fire in Mato Grosso, Pará, Maranhão and Tocantins, as well as areas not previously examined in Amazonas and Amapá states. Recent digitization of the RADAM Brasil project permitted comparison among RADAM's parataxonomists' observations, previous botanical collections and our prospecting. Mapping on soils and vegetation types enabled us to hypothesize a set of ecological preferences. Wild peach palm is best adapted to Ultisols (Acrisols) in open forests across the Arc of Fire and westward into the more humid western Amazonia. Populations are generally small (fewer than 10 plants) on slopes above watercourses. In northern Mato Grosso and southern Pará soybean fields and pastures now occupy numerous areas where RADAM identified wild peach palm. The controversial BR-163 Highway is already eroding wild peach palm as deforestation expands.

**Conclusions/Significance:**

Many of these populations are now isolated by increasing forest fragmentation, which will lead to decreased reproduction via inbreeding depression and eventual extinction even without complete deforestation. Federal conservation areas are less numerous in the Arc of Fire than in other parts of Brazilian Amazonia, although there are indigenous lands; these conservation areas contain viable populations of wild peach palm and require better protection than they are currently receiving. *Ex situ* conservation of these populations is not viable given the relative lack of importance of domesticated peach palm and the difficulty of maintaining even economically interesting genetic resources.

## Introduction

The expansion of the agricultural frontier across southern Amazonia is a well-known phenomenon, with yearly reports on the rate of deforestation and the approximate number of square kilometers cleared and burned to make way for agriculture [1]. The upland forests in this so-called Arc of Fire form a complex mosaic, with dry forests in the transition from the seasonally dry Cerrado (central Brazilian savanna) to open forests with and without palms, and finally to denser forests where rainfall is high and evenly distributed [2], [3]. The soils that support these forests are also varied but are predominantly Ultisols (Acrisols) on and along the edges of the Brazilian Shield; these are slightly richer in nutrients than the Oxisols (Ferralsols) within the sedimentary basin [4]. It is important to note that these forests are relatively new, having expanded to their present range during the Holocene, which started about 11,000 years ago [5]. This is important because it is the same time frame as the arrival of humans, their adaptation to these changing ecosystems, and the development of indigenous agriculture [6]. During the last thousand years, the southwestern section of this area saw the rise and European-mediated collapse of complex agricultural societies in northern Bolivia [7], eastern Acre [8] and the upper Xingu River basin of Mato Grosso [9].

What is less well known is that the Arc of Fire is home to the wild relatives of several native South American crops. Peach palm (*Bactris gasipaes*) and annatto (*Bixa orellana*) may have been domesticated in SW Amazonia [10], [11]. Piperno and Pearsall [6] suggest that several annual crops were domesticated in SW Amazonia, including manioc (*Manihot esculenta*), yautia or cocoyam (*Xanthosoma sagittifolium*), and jack bean (*Canavalia plagiosperma*). Recent work confirms that wild manioc, subsp. *flabellifolia*, was domesticated in SW Amazonia and became subsp. *esculenta* [12], and that the wild subspecies is distributed throughout the drier transitional forests of the Arc of Fire [13]. Bianchetti [14] proposes that one of the possible origins of the Tabasco hot pepper (*Capsicum frutescens*) is in SW Amazonia near the Andean foothills. Several other domesticated fruit crops probably originated along the southern edges of Amazonia also [15].

Recent work supports the hypothesis of Huber [10] that peach palm may have been domesticated in the western part of the Arc of Fire [16], [17], and the occurrence of one of the wild types ancestral to peach palm (*B. gasipaes* var. *chichagui* type 1) was recently confirmed in the open forests of SE Amazonia [18], as well as in SW Amazonia [19] and W Amazonia [20]. During the domestication process, the peach palm was dispersed from SW Amazonia in two directions: northeastward down the Madeira and Amazon Rivers to the Atlantic seaboard; northwestward out of the upper Madeira River basin and into the Ucayali River basin, from where it was dispersed throughout western Amazonia, northern South America and into Central America [17]. Peach palm became a major starchy staple in W Amazonia, NW South America and S Central America [21], becoming the premiere Neotropical palm domesticate [22]. Several governmental research institutions in peach palm's range have worked intensively to develop information for fruit and heart-of-palm farmers and agribusinesses within the region [21], [23], with a strong emphasis on the use and conservation of the crop's genetic resources. Only recently, however, have the wild populations of peach palm received much attention.

Numerous projects within the Program on Brazilian Biodiversity (ProBio, Environment Ministry of Brazil) recently examined the distribution of the wild relatives of several important native crops [24]. The present report examines the case of wild peach palm and relates its distribution to soils and vegetation types. Additionally, the Brazilian Institute for Geography and Statistics (IBGE - Instituto Brasileiro de Geografia e Estatística) recently digitized the first modern mapping exercise done in Brazilian Amazonia: the RADAM Brasil project (Projeto Radar na Amazônia Brasil) used side-viewing airborne radar to map the region and followed up with 3,131 ground surveys that included rapid botanical inventories of economic plants [25]. A comparison among our ProBio survey, RADAM's information and previous collections is presented in terms of wild peach palm. Conservation is discussed in light of expanding deforestation, lack of resources for *ex situ* conservation throughout the developing world, and climate change.

## Methods

### Taxonomy and distribution of peach palm

**Figure 1:**
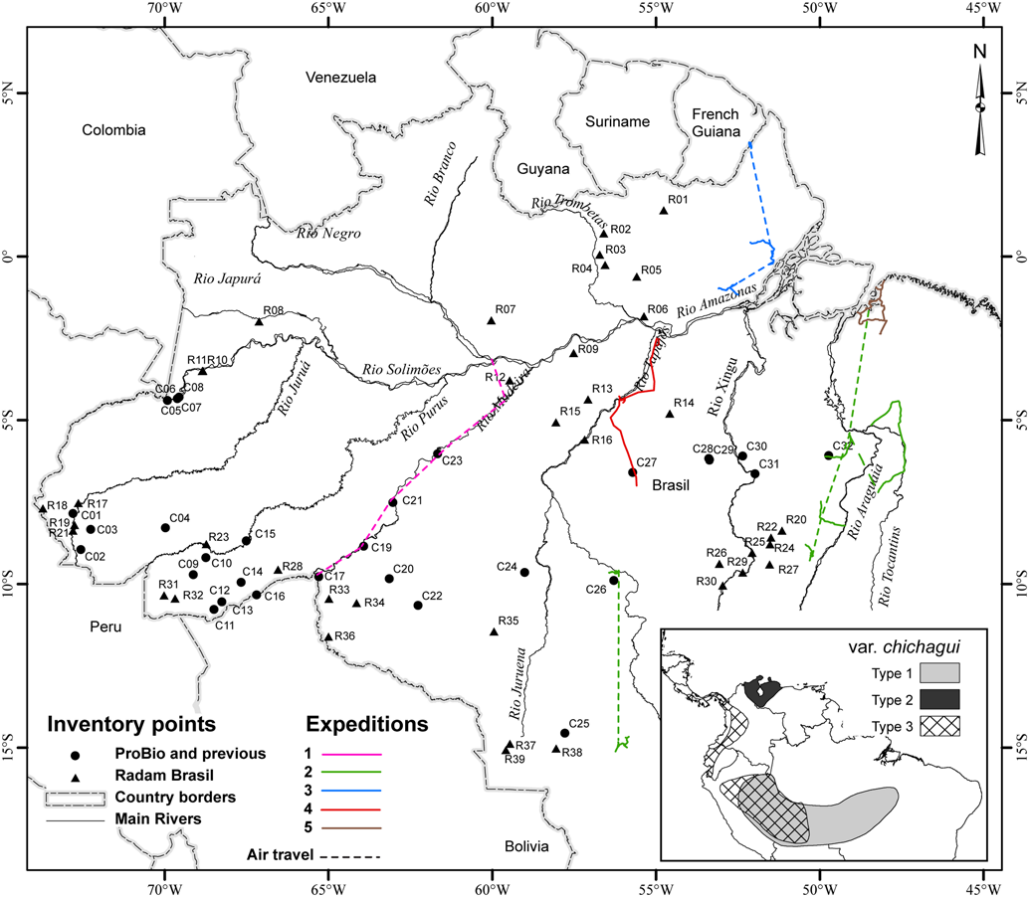
Brazilian Amazonia's river system and state boundaries, with location of known collections and confirmed observations of *B. gasipaes* var. *chichagui*, including ProBio observations (all Confirmed-number – Cn), plus RADAM Brasil observations of wild peach palm (all RADAM-number – Rn). See Appendix S1 for data point identification. Colored lines represent the five ProBio expeditons, with both terrestrial routes and air connections; Expedition 2 (green) also had air travel between state capitals that is not shown. Inset: approximate distributions of the three types of var. *chichagui*.

**Figure 2:**
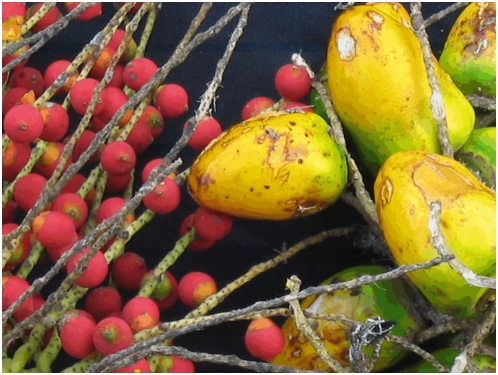
Contrast between wild (*Bactris gasipaes* var. *chichagui* type 1 – Left) and cultivated (*B. gasipaes* var. *gasipaes* Pará landrace – Right) fruits near Parauapebas, Pará, Brazil. Credit: Evandro Ferreira.

Peach palm is the only domesticated palm in the Neotropics [22]. Until recently, it was considered a cultigen [11] – a cultivated species with no known wild relatives. Research and development of peach palm over the last 30 years changed this evaluation [21]. The most recent revision of *Bactris* united all wild populations into a single variety, *B. gasipaes* var. *chichagui* (H. Karsten) A. J. Henderson, and all domesticated populations and landraces into *B. gasipaes* var. *gasipaes* [26]. Within var. *chichagui*, Henderson proposed three types, based principally upon fruit dimensions, without describing their distribution or attributing synonyms to each type; approximate distributions are presented in Figure 1 (inset). Both type 1 and type 2 have very small fruit (0.5 to 2 g), while type 3 has small fruit (3 to 10 g); var. *gasipaes* has fruit that range in mass from 10 to 200 g (Figure 2). We adopt Henderson's classification in this study.

Our work in Brazilian Amazonia identified types 1 and 3 as occurring along the western side of the Madeira River in SW Amazonia, allowing the suspicion that Huber [10] may have considered these the same entity when describing *Guilielma microcarpa* (now var. *chichagui*) along the adjacent Purus River. The material that Huber saw near Pucallpa, Peru, was probably type 3 [19]. Both type 1 and type 3 fruits were found near Pozuzo, Peru, the type locality of *Martinezia ciliata* Ruiz & Pavón (EF, pers. obs.; *M. ciliata* is now var. *gasipaes*.). These observations suggest that the original Ruiz and Pavón description may have included both types. Hence, these early botanists may have had wider species concepts than originally thought, without, however, realizing the synonymy with the original *B. gasipaes*, although Huber hypothesized that a cross between *G. microcarpa* and *G. insignis* (now var. *gasipaes*) may explain the origin of *B. gasipaes*, which he recognized as *G. speciosa*. Huber's hypothesis was based on fruit morphology and Henderson's revision now makes it superfluous, as Huber's entities are now all part of the same species.

The major remaining question is where the peach palm was domesticated. As mentioned, Huber [10] suggested SW Amazonia, and recent work lends support to this idea [16], [17], [27] without, however, evaluating the hybridization hypothesis. Morcote-Rios and Bernal [28] hypothesize that it was domesticated in the range of type 3 in NW South America, especially Colombia, based on archaeological information. Mora Urpí [29] hypothesizes several domestication events in various locations within the distribution of var. *chichagui* type 3 and a recent analysis provides tantalizing relationships that may support this hypothesis [30]. We will not attempt to answer this question here, but it is clear that the var. *chichagui* populations across southern Amazonia, especially in the southwest, are important and worthy of conservation.

### The ProBio expeditions

The project financed by ProBio was designed to determine the distribution of var. *chichagui* south of the Amazon River, especially along the transition between the Cerrado and the humid forest. Five prospecting expeditions were carried out in 2005 (Figure 1). The first expedition (March 2005) concentrated on the Madeira River, the connection between the hypothesized origin of peach palm as a domesticate and the major population centers of pre-Columbian Amazonia on the Amazon River [6]. The second expedition (April 2005) concentrated on the Arc of Fire, from central Mato Grosso through southeastern Pará to western Maranhão. The third expedition (April 2005) concentrated on northeastern Pará State to identify the eastern limits of var. *chichagui* type 1. The fourth expedition (May 2005) concentrated on the extreme northeast of Amazonia, the state of Amapá, to try to identify possible wild populations. The final expedition (May 2005) concentrated on the BR-163 that cuts south through central Amazonia from Santarém, Pará, to Cuiabá, Mato Grosso, in order to attempt to identify the northern limit of the var. *chichagui* type 1 populations found across the Arc of Fire.

The first expedition traveled by plane, stopping at each municipal seat, where the two-man team interviewed people at town markets and the local extension agency about wild peach palm. When wild peach palm was reported, it was confirmed with visits to see plants. The other expeditions traveled by plane to target areas and then by car along major and secondary highways. At each urban center, the two-man teams interviewed people at town markets and the local extension agency; in Alta Floresta, the team interviewed people at the CEPLAC (National Cacau Promotion Commission) station as well. Between urban centers, the teams observed the landscape and stopped at candidate areas for wild peach palm, both primary forest (principally in Mato Grosso) and secondary forest; when found, plants and fruit bunches were photographed. The teams avoided entering private land unless wild peach palm was observed, in which case permission was obtained to photograph. All wild peach palm was geo-referenced (see Appendix S1).

### The RADAM dataset

The RADAM Project started in 1970 and in 1975 was expanded to cover the rest of Brazil outside of Amazonia [25]. With the digitization of this data set by IBGE, it became possible to easily transfer information into geographic information systems to compare the extensive RADAM data set with others, such as the ProBio-financed peach palm data set. This comparison is both enlightening and somewhat frustrating because peach palm was not considered a major economic species by RADAM.

The 3,131 ground surveys included a soils characterization pit and a rapid survey of the vegetation along a transect near the pit [25]. More than 750 timber species were identified, with attempts at applying Latin names. Informal observations were made of palms, dicot fruit species and a few other economically useful species, with no Latin names. Wild and sometimes cultivated peach palm is included.

We use the soils and vegetation characterizations to identify apparent adaptations of var. *chichagui* across the Arc of Fire. Given that there are only 3,131 ground surveys in the 5 million square kilometer Brazilian Amazon basin, precision identification of soils and vegetation types is only possible for the RADAM data records and even these may not be precise given local environmental heterogeneity within the area of the botanical survey. We feel that the vegetation types are generally more reliable than the soils, but by examining soils at the class level rather than the great group level we expect greater reliability. Nonetheless, it is clear that this is only a first approximation and further work is necessary to refine our interpretation.

## Results and Discussion

### The ProBio expeditions for wild peach palm

The first expedition proceeded along the Madeira River, Amazonas, from Borba south to Porto Velho, Rondônia. Throughout the Madeira basin the Pará landrace of cultivated peach palm is common, confirming Mora Urpí's [29] hypothesis that the Madeira River connects the Pará landrace with the possible origin of the domesticate in SW Amazonia. In Borba, there is a large plantation of exotic peach palm (spineless germplasm from the Pampa Hermosa landrace, around Yurimaguas, Peru, is used in the heart-of-palm agribusiness [21]), and some has been managed for seed production. Consequently, exotic fruit are also coming into the market, but do not attract local consumers, who think these fruit are too starchy compared to their oilier Pará landrace fruit. At Manicoré the team was informed that wild peach palm occurred 50–60 km south of town, but they were unable to confirm this. In Porto Velho, the team found type 3 *chichagui*, with fruit weighing 5–6 g. Similar fruits were found at Humaitá, Amazonas. These two finds are the eastern-most records of type 3 *chichagui* and place it in an appropriate region to have participated in the domestication of peach palm.

**Figure 3:**
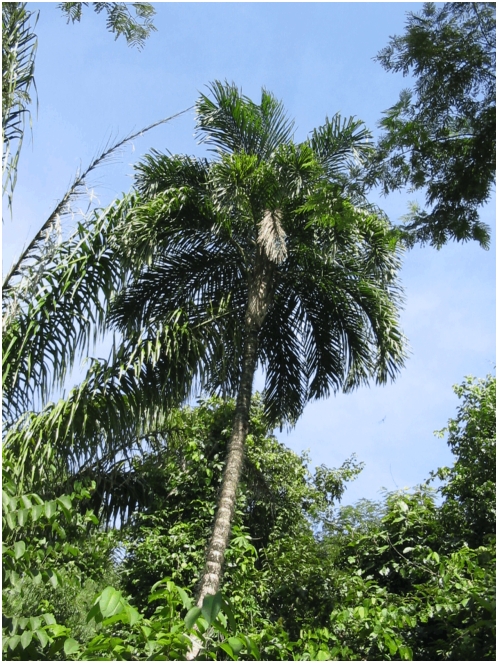
*Bactris gasipaes* var. *chichagui* type 1 in second growth vegetation near Alta Floresta, Mato Grosso, Brazil. Credit: Evandro Ferreira.

The second expedition started in Cuiabá, Mato Grosso, and attempted to find the populations of *B. gasipaes* described from this area under the name *Guilielma mattogrossensis* Barbosa Rodrigues [31] in the region of the Chapada dos Guimarães, a complex of hills just north of Cuiabá. The whole region has been dramatically altered since Barbosa Rodrigues' visit, with pastures and second growth vegetation predominating in and around the Chapada. The approximate type locality is also partially occupied by the inundation area of the Manso River hydroelectric dam. Without finding Barbosa Rodrigues' *G. mattogrossensis*, the team flew north to Alta Floresta in the epicenter of current deforestation in Mato Grosso's rapidly expanding agricultural frontier. The trip north went over significantly altered landscapes, with pasture and field crops, principally soybean, alternating with second growth. Around Alta Floresta the amount of fragmented forest was significant, with land use change evident on all sides. In the area of Alta Floresta, however, numerous populations of type 1 *chichagui* were observed in forest fragments and second growth (Figure 3).

In southeastern Pará the team found small, fragmented populations in Parauapebas, Pará, south of Marabá, which expands the eastern limits of type 1 *chichagui* a couple of hundred kilometers (Figure 1), but none as they advanced south and westward through Santana de Araguaia, Redenção and Conceição de Araguaia, the more active and consolidated part of Pará's cattle ranching frontier. Although this whole region once contained open forests with palms, apparently appropriate for type 1 *chichagui* and similar to the forest fragments in Parauapebas and São Felix do Xingu, not a single plant was observed. The region is now dominated by pasture, some of which is very well managed, and by second growth vegetation, suggesting that any populations that may once have existed are now extinct even in second growth near roads. In Ourilândia do Norte, extreme southern Pará, Salm [32] did not find type 1 *chichagui* in undisturbed areas that we think appropriate for occurrence of the species (see below). The team returned north and went to Imperatriz, Maranhão, and then south through the Cerrado into northern Tocantins, without observing wild peach palm in gallery forests. The cultivated peach palm that was observed was reported to have been recently introduced from Belém, Pará. This part of the expedition was designed to confirm that type 1 *chichagui* does not grow south of the Araguaia River, even though there are some ecosystems where it might be expected, i.e., the gallery forests.

The third expedition went to extreme northeastern Pará. The Zona Bragantina is the oldest agricultural frontier in Amazonia and now occupied almost exclusively by second growth vegetation. As expected, Pará landrace peach palm was abundant, but there were no signs of wild peach palm, again suggesting that var. *chichagui* is limited by the Tocantins-Araguaia River system in the east, as well as by the Araguaia River in the south.

The fourth expedition scouted the extreme northeast of Brazil with the idea that type 2 *chichagui* might extend along the northern flanks of the Guiana shield, even though it has not been reported from any area east of northwestern Venezuela [26]. Amapá State has one of the lowest rates of deforestation in Amazonia, although the cerrado of Amapá is starting to attract the attention of soybean farmers. Pará landrace peach palm is common in the homegardens in Amapá. In Oiapoque, close to the frontier with French Guiana, a single plant, with a thinner stem, smaller leaves and small fruit bunches was found, but the bunch was dry and no seeds were found on the ground. Neighbors claimed that the fruit is so small that it cannot be sold, but this is not enough to claim to have found wild peach palm in Amapá. During a previous expedition to the Serra do Navio, west of the cerrado, no wild peach palm were observed (EF, pers. obs.).

The final expedition went to the BR-163, the Santarém (Pará) to Cuiabá (Mato Grosso) highway, now a center of world conservation attention because it will soon be paved and connect the soybean fields of Mato Grosso with Cargill's port in Santarém. The team also scouted the lower Tapajós River as far as Itaituba without finding wild peach palm. Along the highway south of Santarém, the team found wild peach palm in Novo Progresso (Figure 1), about halfway to the frontier with Mato Grosso. Although the federal government is attempting to plan the occupation of the BR-163 to slow the rapidly expanding agriculture frontier, the team found an increasingly fragmented open forest, interspersed with soybean fields and pastures, with considerable new second growth.

### The RADAM data set

The RADAM Amazonian data set contains 3,131 sites where vegetational surveys were executed. Of these, 42 sites mention the names ‘pupunha’, ‘pupunharana’ or ‘pupunha brava’; these are usually translated as peach palm, false peach palm and wild peach palm, respectively.

The name ‘pupunharana’ is the most complicated, as it has been applied to various other palms in different parts of Amazonia and 35 data points mention this taxon. In Acre it may be applied to *Aiphanes aculeata* and even to the stemless *Bactris acanthocarpa*, as well as to *B. gasipaes* var. *chichagui*. Hence, in Acre it is difficult to determine if the RADAM sites always represent var. *chichagui*, but we assume they do because of its local abundance (EF and CC, pers. obs.). In Central Amazonia, ‘pupunharana’ is often used to name *Syagrus cocoides* and *S. inajai*; the latter species is also called ‘pupunha de porco’ (pig's peach palm) north of Manaus. No var. *chichagui* has yet been found north of Manaus in Amazonas state (CC, pers. obs.). Along the Trombetas River between Pará and Amazonas states, ‘pupunha brava’ is applied to *Syagrus cocoides*, which is locally abundant, and no var. *chichagui* have been observed to date (Ires Miranda, INPA, pers. com., 2007). Given the lack of var. *chichagui* in the Trombetas River basin and north of Manaus, seven of the RADAM sites (Figure 1, sites R01–R07) are extremely unlikely to be var. *chichagui*. Several other sites will require future field work, especially those just south of the Amazon River (Figure 1, sites R09, R12), which are also likely to be *Syagrus*, and one site just north of the Solimões River on the Japurá River (Figure 1, site R08), outside of the range of the two *Syagrus* mentioned [33].

In Acre, Rondônia and western Mato Grosso states, the RADAM data set is in good agreement with our confirmed observations of var. *chichagui* (Figure 1), so we can be reasonably sure of this information. In southern Pará state, RADAM botanists presumably found var. *chichagui* in areas adjacent to those in which our expeditions failed to find it (Figure 1, sites R20, R22, R24, R25, R27), suggesting that land use change has indeed eliminated populations in that area. In central Pará state, four RADAM sites are adjacent to the TransAmazon Highway (BR-240) and to the BR-163 (Figure 1, sites R13–R16), and extend the possible distribution of var. *chichagui* further north, into soils and forests that are less typical of those where most other populations are found (see below). In western Amazonas state, two RADAM sites are located in São Paulo de Olivença, not far from the group of sites with recent confirmed observations (Figure 1, sites R10–R11).

In general, the RADAM data set is in quite good agreement with our confirmed observations, except in Central Amazonia north of the Amazon River, and helps us to understand the relationship of this variety with original vegetation types and soils. The somewhat dubious sites mentioned above will be targeted for future fieldwork.

### Relations with original vegetation types and soils

**Figure 4:**
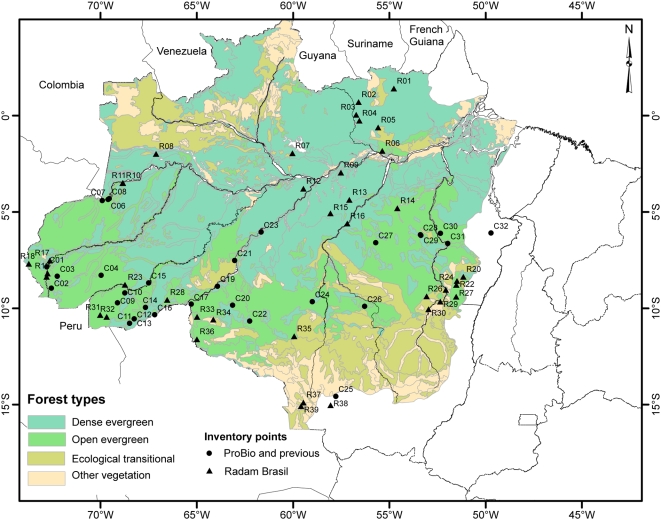
Confirmed distribution of *Bactris gasipaes* var. *chichagui*, types 1 and 3 (points with Cn – Confirmed-number) and RADAM data set (points with Rn – RADAM-number) in Brazilian Amazonia, against a background of general vegetation types. See Appendix S1 for data point identification.

The majority of the ecosystems where var. *chichagui* type 1 has been found to date are generically classified as open evergreen forest (Figure 4). Pires and Prance [2] discriminated between open forest with palms and without palms, but mentioned that open forest with palms is much more common than without. The current Brazilian vegetational classification system [3], [34] does not discriminate between these two forest types. Moderate stature palms, such as var. *chichagui*, cannot generally attain the canopy of dense forests [35], [36], so their occurrence in open forests is understandable. Cultivated peach palm ceases to fruit when the second-growth forest canopy closes over it in areas where dense forests are the norm [37], but this is probably not the case for var. *chichagui* in open forests.

Variety *chichagui* type 1 has been observed in dense forests in the Serra do Moa, part of the Serra de Divisor National Park, western Acre (EF, pers. obs.). Hence, the relation with open forests is not absolute, raising the possibility that some of the RADAM sites in dense forests south of the Amazon River but north of the Brazilian Shield may be var. *chichagui* (e.g., Figure 4, sites R12, R13, R15, R16).

**Figure 5:**
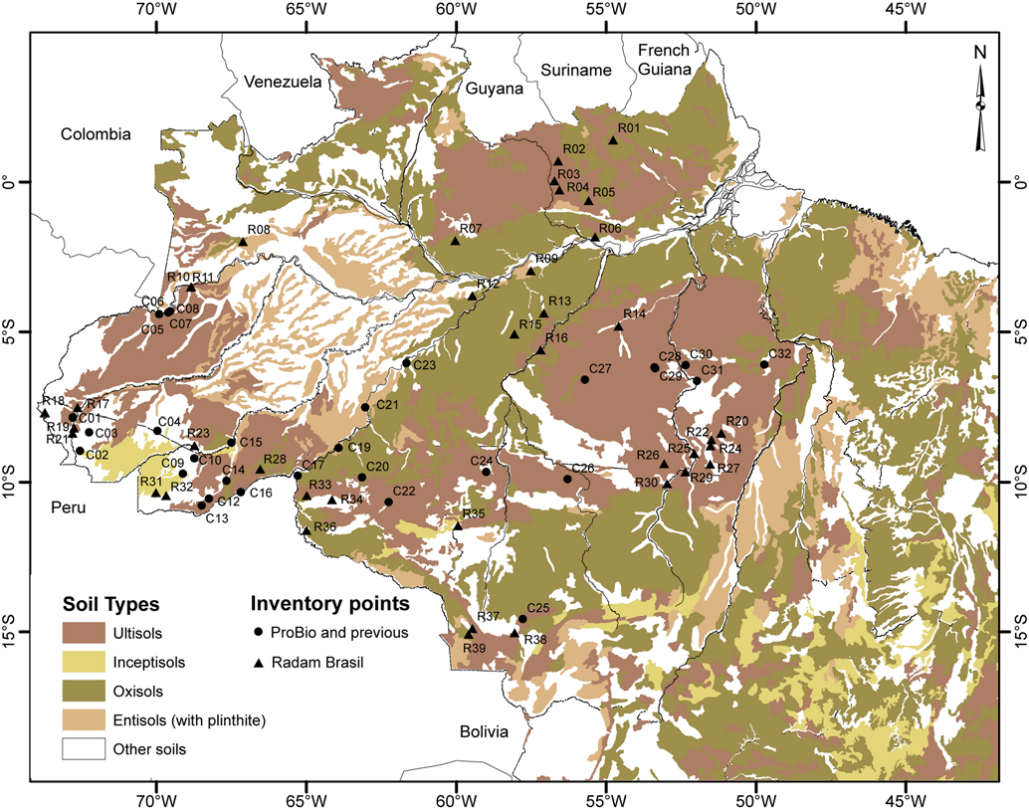
Confirmed distribution of *Bactris gasipaes* var. *chichagui*, types 1 and 3 (points with Cn – Confirmed-number) and RADAM data set (points with Rn – RADAM-number) in Brazilian Amazonia, against a background of soil orders. See Appendix S1 for data point identification.

The relationships of var. *chichagui* type 1 with soils across southern Amazonia are relatively clear also at the soils family level – it is well adapted on Ultisols, termed Acrisols in the FAO system (Figure 5). Among the 31 confirmed observations, 74% are on Ultisols and 10% on even richer Alfisols (Luvisols), the latter in western Acre, while only 10% are on Oxisols (Ferralsols); among the 39 RADAM observations, 44% are on Ultisols, 13% on Alfisols, 23% on Oxisols and 19% on Entisols, again suggesting that the RADAM observations mix var. *chichagui* with other palms. In the Madeira River basin, for example, there are confirmed instances of type 1 on Oxisols, suggesting a different adaptation and reenforcing its presence in denser forests. A clear non-adaptation is to the soils with plinthite (Plinthosols) of the lower and middle Purus and Juruá River basins, with confirmed presence only in the upper basins where Ultisols are common. Another clear non-adaptation is to the Oxisols in the upper Xingu and Tapajós River basins, explaining the absence of type 1 in the Kayapó Indigenous Reserve, Ourilândia do Norte, Pará [32].

The relations of var. *chichagui* type 3 are less clear, certainly because type 3 has not been observed as much. It has been observed on Oxisols in the middle to upper Madeira River basin in Brazil, while in the upper Purus River basin in Acre it is not clear if the observations are on Oxisols or Ultisols, and in extreme western Amazonas (Figure 5, C05, C06, C08) they are on Ultisols. Since most of the Amazonian populations of type 3 are found in Bolivia and Peru, we will need to expand our analysis more widely before asserting soil adaptations.

### Conservation and local extinction

In Amazonia, land use change is an euphemism for deforestation and the expansion of agriculture and pastures. Worldwide, land use change is expected to commit 7 to 24% of vascular plant species to extinction by 2050 [38]. Our observations in the Arc of Fire suggest that wild peach palm has become locally extinct in some parts of its original range, due directly to deforestation for agriculture and pasture, and indirectly to deforestation for pasture and the use of fire for subsequent pasture management [18]. However, wild peach palm is unlikely to be committed to extinction by 2050 because of the lower rate of deforestation in western Amazonia, although eastern Amazonia is more problematic.

In the Arc of Fire there are some site-specific situations that permit escape from immediate extinction; all depend upon the fact that palms are often left standing when other trees and brush are cut. Isolated plants or small populations can survive (1) in areas with very rough relief [18], where deforestation may occur but where it is too difficult or impossible to establish a new land use, (2) in areas where natural vegetation is left to protect stream and river edges [18], and (3) in areas along the edges of roads or even along fence lines, where clearing of brush is infrequent or less rigorous [19]. All of these situations are subject to annual fires for pasture management, but they allow some survival, at least temporarily.

In general, isolated individuals are functionally extinct, even though alive, because they are unable to reproduce except via self-pollination and their progeny are likely to suffer inbreeding depression if they manage to grow in the disturbed environment. In domesticated peach palm cross-pollination is the norm [39], although high levels of selfing can be forced with controlled pollination [40]. Peach palm is pollinated principally by small Curculionid weevils with 400–500 m flight autonomy [21], and wild peach palm is probably dispersed by both birds and seed hoarding mammals, although no direct observations have been published to date. Small populations are also seriously compromised in evolutionary terms, as the degree of inbreeding will increase continually unless birds bring new seeds from other nearby populations. This can be evaluated in terms of the effective size of the population, the number of individuals that effectively participate in annual reproduction.

The effective size of a population of wild peach palm that would be evolutionarily viable is unknown, but a recent study of the mating system in three populations of cultivated peach palm offers useful information [39]. In each population, eight open-pollinated progenies had been pollinated by between 9 and 20 pollen donors, helping to explain the 95–99% outcrossing rates and very low estimates of self-fertilization (1–5%). These very high levels of outcrossing are similar to those reported in other palms, e.g., in *Astrocaryum mexicanum* [41] and *Euterpe edulis* [42], [43], both wild species. Estimates of inbreeding in cultivated peach palm ranged from 13 to 14.5%, most of which was of biparental origin, meaning crossing among sibs [39]. This level is slightly higher than in *E. edulis* [42], [43], probably due to the high frequency of sibs in cultivated populations [22], [39]. Based on this analysis, estimates of effective population number (Ne) ranged from 26 to 30 in peach palm [39], with the caveat that this is for a set of cultivated populations with relatively high census numbers. Effective numbers tend to be about 10% of census numbers in outcrossing species [44], so a population of 250–300 plants would be necessary to keep inbreeding relatively low, but as many as 5000 plants might be necessary to guarantee evolutionary viability [44], [45], especially considering ongoing climate change that is likely to demand greater flexibility. A study similar to Rodrigues' [39] in a set of var. *chichagui* populations in the Arc of Fire would provide important information to continue planning for *in situ* conservation.

These effective sizes are larger than any population of wild peach palm observed to date. Small populations of 5 to 10 individuals are the norm in Rondônia and SE Pará states [18], [19]; in both cases these small populations were in disturbed areas and separated by several kilometers from the next population. Evandro Ferreira (pers. obs.) observed densities lower than 1 palm/ha in primary forests in Acre state. However, in the municipality of Alta Floresta, Mato Grosso state, we observed 20 individuals in 2.5 ha in intact open forest conserved on the CEPLAC experiment station, so there appear to be areas of locally high abundance, at least in the range of var. *chichagui* type 1.

The first implication of these small populations is that they depend upon gene flow from neighboring populations to maintain their genetic variability and evolutionary flexibility. In fragmented landscapes, the requisite gene flow is less likely to occur [46]. Hence, even though numerous plants and small populations are temporarily surviving in the Arc of Fire, these do not represent viable *in situ* conservation units, since their future is likely compromised by reproductive limitations and increasing inbreeding.

**Figure 6:**
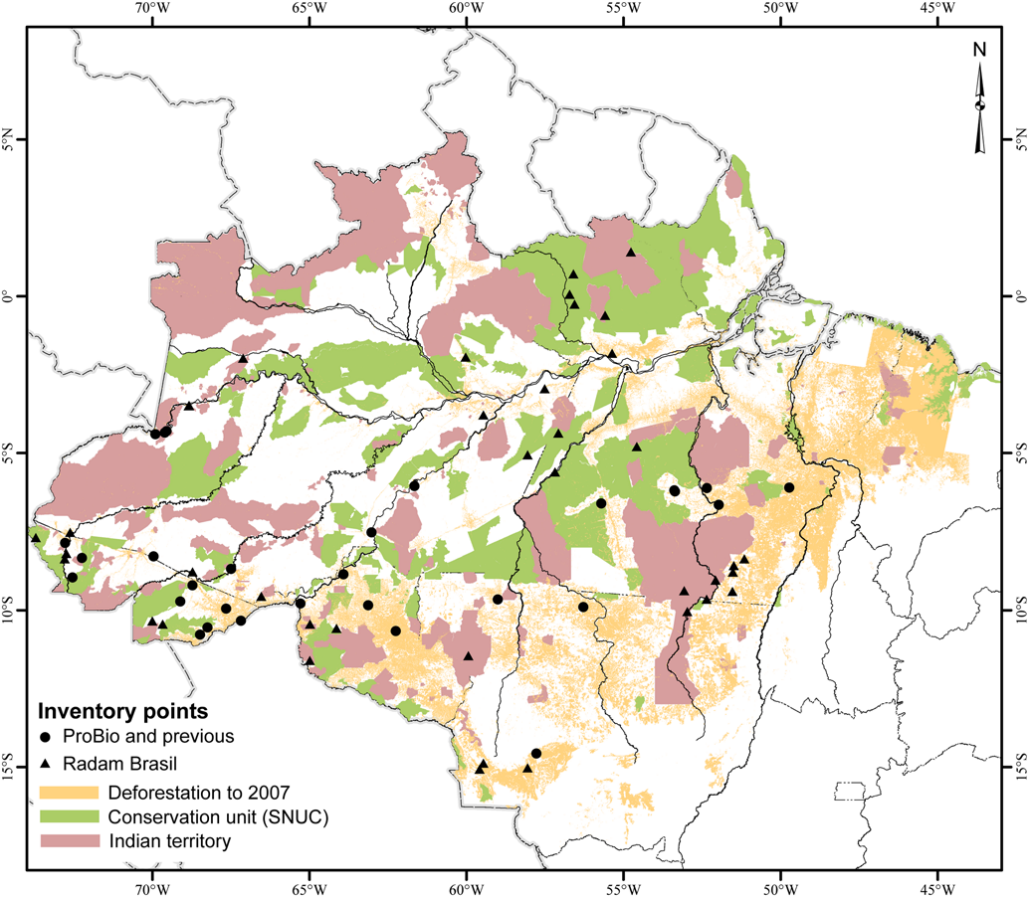
Distribution of *Bactris gasipaes* var. *chichagui* in Brazilian Amazonia, against a background of the Brazilian National System of Conservation Units (SNUC) and Indigenous Lands, as of 2008, with deforestation data from 2000–2007 (INPE-PRODES. Deforestation in Amazonia Legal. http://www.obt.inpe.br/prodes/). Only one third of the SNUC lands have personnel and infrastructure, and none have sufficient personnel to guarantee against invasions.

That leaves the Brazilian National System of Conservation Units (SNUC) and Indigenous Lands for *in situ* conservation. Unfortunately, the Arc of Fire is not well represented within the SNUC, although the federal government has recently decreed several “paper” conservation units, but there are numerous Indigenous Lands (Figure 6). In fact, the latter tend to have better integrity than the former [47], as indigenous peoples have numerous allies to help protect their lands, while the SNUC lands tend to be uninhabited, although some new Sustainable Development Reserves and Extractive Reserves have been created over the past two decades. Most of the SNUC areas and Indigenous Lands are large enough to have evolutionarily viable meta-populations of wild peach palm, which will survive as long as the integrity of the area is maintained. The only Indigenous Land with current information about wild peach palm is the Kayapó reserve in Ourilândia do Norte, extreme southern Pará, where Salm [32] did not find var. *chichagui*. The large Xingu reserve in northeastern Mato Grosso apparently does not contain var. *chichagui* either. In terms of forest cover, it is appropriate, but not in terms of soils (Figures 4 and 5). Nonetheless, other Indigenous Lands certainly do contain wild peach palm, and this can be verified in the future.

In the western part of wild peach palm's distribution in Brazilian Amazonia, especially in Amazonas and Acre states, the conditions for *in situ* conservation are more favorable than in the central and eastern parts. In Acre, the Chico Mendes and Upper Juruá Extractive Reserves, with 900,000 ha and 500,000 ha, respectively, both contain populations of wild peach palm and both are certainly evolutionarily viable. The Mamoadate Indigenous Land, with 313,000 ha, contains populations of wild peach palm and wild plants are even tolerated in swidden plots [48]. Nonetheless, this generally favorable situation is only temporary, as agricultural expansion is already accelerating in the region and will continue with the paving of the BR-364 into western Acre and the BR-319 in Amazonas state. Both highways are in the distribution of wild peach palm and, in fact, these populations may be more important than the central and eastern populations because they are more closely related to the domestication of peach palm [17].

In the central part of wild peach palm's distribution in Brazil, essentially northeastern Mato Grosso and southwestern and western Pará states, the conditions for *in situ* conservation are less favorable, as the forests are being fragmented rapidly. Along the BR-163, which connects Santarém, Pará, a major port on the Amazon River, with Cuiabá, the capital of Mato Grosso, fragmentation is being driven by timber high-grading, followed by clearing for pasture, and finally expansion of soybean. The BR-163 will be paved soon as part of Brazil's new Plan to Accelerate Economic Growth (Plano de Aceleração do Crescimento) in order to move soybean from northern Mato Grosso to the world market via Santarém. Laurance et al. [49] examined the mid-term impact of this road within the previous government's development plan, which stimulated the Brazilian Ministry of the Environment to establish numerous conservation units along this highway in 2005 [50]. While it looks good on paper, it remains to be seen whether these areas will survive the agricultural expansion now at full throttle in the region.

This raises the issue of *ex situ* conservation, especially as peach palm is increasingly important for its heart-of-palm [21]. The outlook for this type of conservation is not favorable, however, as numerous *ex situ* collections of peach palm have lost their funding because they have not presented favorable cost/benefit ratios, others are under-funded and some have been abandoned [51]–[54]. At first glance, this is curious, given the expansion of the hearts-of-palm agribusiness in Latin America. The reasons are clear, however: the hearts-of-palm expansion depends upon genetic resources from a single landrace, the Pampa Hermosa near Yurimaguas, Peru [21], [52]; the major collections contain numerous landraces with no commercial demand, as well as some samples of wild peach palm, hence agribusiness is uninterested in supporting them because they are not directly pertinent – in fact, at this early stage in the expansion, no agribusiness is willing to support conservation. Hence, the idea of conservation-through-use that is central to the Convention on Biological Diversity is not applicable to peach palm *ex situ* collections at this stage of its economic expansion.

For a tree species, like peach palm, it is now appropriate to include climate change in analyses for conservation, since significant change is expected within the 30 to 100 year life span of a clump of peach palm. In Amazonia, where El Niño events are important, the Hadley Centre model [55] is considered to be the most appropriate for planning. This model predicts savannaization in eastern Amazonia before mid-century, with subsequent expansion westward until all forest is savanna by 2080. The principal implication is that even large SNUC areas and Indigenous Lands will become fragmented and savannaized.

Using a less severe Hadley Centre model, Miles et al. [56] predict that only 20 to 30% of a set of 69 angiosperm species with variable distributions in Amazonia will have viable populations across southern Amazonia at the end of the century in a business-as-usual scenario, while 60 to 70% may have viable populations if human society works hard to reduce carbon emissions immediately. Wild peanut species across the savannas south of Amazonia are also expected to suffer, with as many as 31 of 51 species going extinct in a business-as-usual scenario [57]. No other studies have included native Amazonian crops and their wild relatives to date, but we can predict that survival of other wild relatives of native South American crops found in the Arc of Fire will depend upon their ability to survive in savannas or in gallery forests, ecosystems in which we failed to find wild peach palm.

## Supporting Information

Appendix S1 Full data set developed and used in project. A. Location of ProBio and previously described observations of wild peach palm (*Bactris gasipaes* var. *chichagui*) in Brazilian Amazonia, with soil types. B. Location of Projeto Radam observations of possible wild peach palm in Brazilian Amazonia, with soil types.
(0.10 MB DOC)

## References

[1] Fearnside PM (2006). Desmatamento na Amazônia: Dinâmica, impactos e controle.. Acta Amazonica.

[2] Pires JM, Prance GT, Prance GT, Lovejoy TE (1985). The vegetation types of the Brazilian Amazon.. Key environments: Amazonia.

[3] Veloso HP, Rangel Filho ALR, Lima JCA (1991). Classificação da vegetação brasileira, adaptada ao sistema universal.

[4] Jordan CF, Prance GT, Lovejoy TE (1985). Soils of the Amazon rainforest.. Key environments: Amazonia.

[5] van der Hammen T, Hooghiemstra H (2000). Neogene and Quaternary history of vegetation, climate, and plant diversity in Amazonia.. Quaternary Science Reviews.

[6] Piperno DR, Pearsall DM (1998). The origins of agriculture in the lowland Neotropics.

[7] Erickson C (2000). An artificial landscape-scale fishery in the Bolivian Amazon.. Nature.

[8] Pärssinnen M, Ranzi A, Saunaluoma S, Siiriäinen A, Pärssinnen M, Korpisaari A (2003). Geometrically patterned ancient earthworks in the Rio Branco region of Acre, Brazil: New evidence of ancient chiefdom formations in Amazonian interfluvial terra firme environment.. Western Amazonia - Multidisciplinary studies on ancient expansionistic movements, fortifications and sedentary life.

[9] Heckenberger MJ, Russell JC, Toney JR, Schmidt MJ (2007). The legacy of cultural landscapes in the Brazilian Amazon: implications for biodiversity.. Philosophical Transactions of the Royal Society B.

[10] Huber J (1904). A origem da pupunha.. Boletim do Museu Paraense Emilio Goeldi, Botanica.

[11] Schultes RE, Stone D (1984). Amazonian cultigens and their northward and westward migrations in pre-Columbian times.. Pre-Columbian plant migration.

[12] Schaal BA, Olsen KM, Carvalho LJCB, Motley TJ, Zerega N, Hugh H (2006). Evolution, domestication, and agrobiodiversity in the tropical crop cassava.. Darwin's harvest – New approaches to the origins, evolution, and conservation of crops.

[13] Allem AC (1999). The closest wild relatives of cassava (*Manihot esculenta* Crantz).. Euphytica.

[14] Bianchetti LB, Walter BMT, Cavalcanti TB (2005). Subsídios à coleta de germoplasma de espécies de pimentas e pimentões do gênero *Capsicum* (Solanaceae).. Fundamentos para a coleta de germoplasma vegetal.

[15] Clement CR, Balée W, Erickson CL (2006). Fruit trees and the transition to food production in Amazonia.. Time and complexity in the Neotropical lowlands: Studies in historical ecology.

[16] Ferreira EJL (1999). The phylogeny of pupunha (*Bactris gasipaes* Kunth, Palmae) and allied species.. Memoirs of The New York Botanical Garden.

[17] Rodrigues DP, Astolfi Filho S, Clement CR (2004). Molecular marker-mediated validation of morphologically defined landraces of pejibaye (*Bactris gasipaes*) and their phylogenetic relationships.. Genetic Resources and Crop Evolution.

[18] Silva JBF, Clement CR (2005). Wild pejibaye (*Bactris gasipaes* var. *chichagui*) in southeastern Amazonia.. Acta Botanica Brasilica.

[19] Clement CR, Aguiar JPL, Arkcoll DB, Firmino JL, Leandro RC (1989). Pupunha brava (*Bactris dahlgreniana* Glassman): progenitora da pupunha (*B. gasipaes* H.B.K.)?. Boletin do Museu Paraense Emílio Goeldi, série Botânica.

[20] Clement CR, Aguiar JPL, Aued-Pimentel S (1999). A pupunha brava (*Bactris dahlgreniana*) no Estado do Amazonas, Brasil.. Acta Botanica Venezuelica.

[21] Mora Urpí J, Weber JC, Clement CR (1997). *Peach palm*. Bactris gasipaes *Kunth*. Promoting the conservation and use of underutilized and neglected crops. 20..

[22] Clement CR, Balick MJ (1988). Domestication of the pejibaye palm (*Bactris gasipaes*): past and present.. The Palm - Tree of life. Biology, utilization and conservation.

[23] Clement CR, Janick J, Paull RE (2008). Peach palm (*Bactris gasipaes*).. The Encyclopedia of Fruit and Nuts.

[24] Coradin L Parentes silvestres das espécies de plantas cultivadas.

[25] RADAM Brasil (1973–1981). Levantamento de recursos naturais: Geologia, geomorfologia, solos, vegetação, uso potencial da terra (22 volumes).

[26] Henderson A (2000). *Bactris* (Palmae)..

[27] Saldías-Paz M, Mora Urpí J, Szott L, Murilo M, Patiño VM (1993). La chonta de castilla (*Bactris gasipaes* H.B.K.): taxonomía y algunos datos económicos en Santa Cruz y su distribución en Bolivia.. IV Congresso Internacional sobre Biologia, Agronomia e Industrialización del Pijuayo.

[28] Morcote-Rios G, Bernal R (2001). Remains of palms (Palmae) at archaeological sites in the New World: A review.. Botanical Review.

[29] Mora Urpí J, Mora Urpí J, Gainza Echeverría J (1999). Origen y domesticación.. Palmito de pejibaye (*Bactris gasipaes* Kunth): su cultivo e industrialización.

[30] Hernández Ugalde JA, Mora Urpí J, Rocha Nuñez O (2008). Diversidad genética y relaciones de parentesco de las poblaciones silvestres y cultivadas de pejibaye (*Bactris gasipaes*, Palmae), utilizando marcadores microsatelitales.. Revista de Biologia Tropical.

[31] Barbosa Rodrigues J (1903). Sertum Palmarum Brasiliensium, ou, relation des palmiers nouveaux du Brésil.

[32] Salm R (2004). Tree species diversity in a seasonally-dry forest: the case of the Pinkaití site, in the Kayapó Indigenous Area, southeastern limits of the Amazon.. Acta Amazonica.

[33] Henderson A, Galeano G, Bernal R (1995). Field guide to the palms of the Americas.

[34] IBGE (1992). Manual técnico da vegetação brasileira.

[35] Kahn F (1986). Life forms of Amazonian palms in relation to forest structure and dynamics.. Biotropica.

[36] Kahn F (1997). The Palms of Eldorado.

[37] Clement CR (1990). Regeneração natural de pupunha (*Bactris gasipaes*).. Acta Amazonica.

[38] van Vuuren DP, Sala OE, Pereira HM (2006). The future of vascular plant diversity under four global scenarios.. Ecology and Society.

[39] Rodrigues DP (2007). Diversidade genética e sistema de reprodução em progênies elites de pupunheira inerme (*Bactris gasipaes* Kunth) com marcadores microssatélites: implicações para o melhoramento do palmito..

[40] Clement CR, Arkcoll DB (1984). Observações sobre auto-compatibilidade em pupunha (*Bactris gasipaes* H.B.K.).. Acta Amazonica.

[41] Eguiarte LE, Pereznasser N, Pinero D (1992). Genetic-structure, outcrossing rate and heterosis in *Astrocaryum mexicanum* (Tropical Palm) - Implications for evolution and conservation.. Heredity.

[42] Gaiotto FA, Grattapaglia D, Vencovsky R (2003). Genetic structure, mating system, and long-distance gene flow in heart of palm (*Euterpe edulis* Mart.).. Journal of Heredity.

[43] Conte R, Reis MS, Mantovani A, Vencovsky R (2008). Genetic structure and mating system of *Euterpe edulis* Mart. populations: A comparative analysis using microsatellite and allozyme markers.. Journal of Heredity.

[44] Frankham R, Ballou JD, Briscoe DA (2004). A primer of conservation genetics.

[45] Namkoong G, Bawa K, Burley J, Shen SS (1991). Forest trees: Managing global genetic resources.

[46] Scariot A (1999). Forest fragmentation: effects on palm diversity in Central Amazonia.. Journal of Ecology.

[47] Nepstad D, Schwartzman S, Bamberger B, Santilli M, Ray D (2005). Inhibition of Amazon deforestation and fire by parks and indigenous lands.. Conservation Biology.

[48] Vivan JL (2008). Análise da tomada de decisão para o uso e conservação de recursos genéticos vegetais em florestas manejadas e sistemas agroflorestais..

[49] Laurance WF, Cochrane MA, Bergen S, Fearnside PM, Delamonica P (2001). The future of the Brazilian Amazon.. Science.

[50] MMA (2006). Plano para Desenvolvimento Regional Sustentável para a Área de Influência da Rodovia BR-163 Cuiabá - Santarém.. http://www.mma.gov.br/estruturas/sca_br163/_arquivos/plano_br_163_texto.pdf.

[51] Clement CR, Coradin L (1995). Case study - Pejibaye (*Bactris gasipaes* Kunth, Palmae) in Brazil.. IPGRI Workshop on field genebank management: Problems and potential solutions.

[52] Clement CR, Weber JC, van Leeuwen J, Domian CA, Cole DM (2004). Why extensive research and development did not promote use of peach palm fruit in Latin America.. Agroforestry Systems.

[53] Clement CR, Lleras Pérez E, van Leeuwen J (2005). O potencial das palmeiras tropicais no Brasil: acertos e fracassos das últimas décadas.. Agrociencias, Montevideu.

[54] van Leeuwen J, Lleras Pérez E, Clement CR (2005). Field genebanks may impede instead of promote crop development: lessons of failed genebanks of “promising” Brazilian palms.. Agrociencia, Montevideo.

[55] Cox PM, Betts RA, Collins M, Harris P, Huntingford C (2004). Amazonian dieback under climate-carbon cycle projections for the 21st century.. Theoretical and Applied Climatology.

[56] Miles L, Grainger A, Phillips O (2004). The impact of global climate change on tropical forest biodiversity in Amazonia.. Global Ecology and Biogeography.

[57] Jarvis A, Lane A, Hijmans RJ (2008). The effect of climate change on crop wild relatives.. Agriculture Ecosystem and Environment.

