# Supplementary material for: Ecological Adaptation of Wild Peach Palm, Its In Situ Conservation and Deforestation-Mediated Extinction in Southern Brazilian Amazonia
Source: PLoS One. 2009 Feb 24;4(2):e4564. doi: 10.1371/journal.pone.0004564 (PMC2642997; doi:10.1371/journal.pone.0004564)
Supplement: Appendix S1 — Full data set developed and used in project. A. Location of ProBio and previously described observations of wild peach palm (Bactris gasipaes var. chichagui) in Brazilian Amazonia, with soil types. B. Location of Projeto Radam observations of possible wild peach palm in Brazilian Amazonia, with soil types. (0.10 MB DOC) [file pone.0004564.s001.doc]

Appendix S1. A. Location of ProBio and previously described observations of wild peach palm (*Bactris gasipaes* var. *chichagui*) in Brazilian Amazonia, with soil type.

| ProBio | Longitude | Latitude | Location Name (State)* | Soil type** | References |
| --- | --- | --- | --- | --- | --- |
| C01 | 72º47'24" | 07º50'60" S | Alto Juruá (AC) | Ultisol | USAID 1984 |
| C02 | 72º33'00" | 08º57'00" S | RESEX Alto Juruá (AC) | Ultisol | Ferreira 133 (HPZ) |
| C03 | 72º15'00" | 08º20'00" S | Natal. Alto Juruá (AC) | Alfisol | Ferreira (obs pess) |
| C04 | 69º58'00" | 08º17'00" S | Igarapé Joaci, Rio Tarauacá (AC) | Alfisol | Ferreira 334 (HPZ) |
| C05 | 69º54'29" | 04º24'21" S | Guanabara II, B Constant (AM) | Ultisol | Clement et al. 1999 |
| C07 | 69º36'27" | 04º21'00" S | Nova Aliança, B Constant (AM) | Ultisol | Clement et al. 1999 |
| C06 | 69º36'27" | 04º21'00" S | Nova Aliança, B Constant (AM) | Ultisol | Clement et al. 1999 |
| C08 | 69º33'01" | 04º18'18" S | Cidade Nova, B Constant (AM) | Ultisol | Clement et al. 1999 |
| C09 | 69º07'00" | 09º43'00" S | Rio Macauã, Sena Madureira (AC) | Inceptisol | Ferreira 395 (HPZ) |
| C10 | 68º44'00" | 09º12'00" S | Rio Caeté, Sena Madureira (AC) | Alfisol | Ferreira 435 (HPZ) |
| C11 | 68º29'24" | 10º46'48" S | Seringal Cachoeira (AC) | Ultisol | Henderson 2000 |
| C12 | 68º15'00" | 10º33'00" S | Seringal Dois Irmãos, Xapuri (AC) | Ultisol | Ferreira 80 (HPZ) |
| C13 | 68º15'00" | 10º33'00" S | Seringal Dois Irmãos, Xapuri (AC) | Ultisol | Bandeira 11 (HPZ) |
| C14 | 67º39'36" | 09º57'00" S | Rio Branco (AC) | Ultisol | USAID 1984 |
| C15 | 67º30'00" | 08º40'48" S | Boca do Acre (AM) | Oxisol | Huber 1904 |
| C16 | 67º11'08" | 10º20'07" S | Plácido de Castro (AC) | Ultisol | USAID 1984 |
| C17 | 65º17'24" | 09º46'48" S | Nova California (RO) | Ultisol | Clement (obs pess) |
| C19 | 63º55'12" | 08º51'00" S | Porto Velho (RO) | Ultisol | ProBio 2005 |
| C20 | 63º08'24" | 09º50'24" S | Ariquemes (RO) | Oxisol | Clement et al. 1989 |
| C21 | 63º01'49" | 07º30'51" S | Humaitá (AM) | Ultisol | ProBio 2005 |
| C22 | 62º15'36" | 10º39'36" S | Ouro Preto do Oeste (RO) | Ultisol | Clement et al. 1989 |
| C23 | 61º39'36" | 06º02'24" S | Manicoré (AM) | Oxisol | ProBio 2005 |
| C24 | 59º00'26" | 09º39'10" S | P.A. Juruena, Cotriguaçu (MT) | Entisol | Vivan (obs pess) |
| C25 | 57º46'39" | 14º33'56" S | Tangara da Serra (MT) | Ultisol | Yuyama (obs pess) |
| C26 | 56º17'07" | 09º53'51" S | Alta Floresta (MT) | Ultisol | ProBio 2005 |
| C27 | 55º42'36" | 06º35'24" S | Novo Progresso (PA) | Ultisol | ProBio 2005 |
| C28 | 53º23'14" | 06º10'45" S | São Félix 1 (PA) | Ultisol | SilvaClement 2005 |
| C29 | 53º21'58" | 06º12'54" S | São Félix 3 (PA) | Ultisol | SilvaClement 2005 |
| C30 | 52º21'01" | 06º06'24" S | São Félix 2 (PA) | Ultisol | SilvaClement 2005 |
| C31 | 51º58'17" | 06º38'10" S | São Félix 4 (PA) | Ultisol | SilvaClement 2005 |
| C32 | 49º43'17" | 06º05'31" S | Parauapebas (PA) | Ultisol | ProBio 2005 |

* States: AC - Acre; AM - Amazonas; MT - Mato Grosso; PA - Pará; RO - Rondônia

** Soil classification at class level according to US Soil Taxonomy, with translation from the Brazilian system according to <http://www.cnps.embrapa.br/sibcs/index.html> (Access 11/12/08)

Appendix S1. B. Location of Projeto Radam observations of possible wild peach palm in Brazilian Amazonia, with soil type.

| Radam | Longitude | Latitude | Location Name (State) | Soils type | References |
| --- | --- | --- | --- | --- | --- |
| R01 | 54º46'12" | 01º25'12" N | Almeirim (PA) | Oxisol | Projeto Radam |
| R02 | 56º36'00" | 00º42'36" N | Oriximiná (PA) | Ultisol | Projeto Radam |
| R03 | 56º43'12" | 00º03'36" N | Oriximiná (PA) | Ultisol | Projeto Radam |
| R04 | 56º33'36" | 00º15'00" S | Oriximiná (PA) | Ultisol | Projeto Radam |
| R05 | 55º35'24" | 00º36'36" S | Óbidos (PA) | Ultisol | Projeto Radam |
| R06 | 55º22'12" | 01º49'12" S | Óbidos (PA) | Oxisol | Projeto Radam |
| R07 | 60º02'24" | 01º56'24" S | Presidente Figueiredo (AM) | Oxisol | Projeto Radam |
| R08 | 67º07'12" | 01º58'48" S | Japurá (AM) | Oxisol | Projeto Radam |
| R09 | 57º30'36" | 02º57'00" S | Boa Vista dos Ramos (AM) | Oxisol | Projeto Radam |
| R10 | 68º49'48" | 03º29'24" S | São Paulo de Olivença (AM) | Ultisol | Projeto Radam |
| R11 | 68º51'00" | 03º29'24" S | São Paulo de Olivença (AM) | Ultisol | Projeto Radam |
| R12 | 59º28'12" | 03º47'24" S | Autazes (AM) | Oxisol | Projeto Radam |
| R13 | 57º04'48" | 04º22'12" S | Itaituba (PA) | Oxisol | Projeto Radam |
| R14 | 54º34'48" | 04º48'00" S | Altamira (PA) | Ultisol | Projeto Radam |
| R15 | 58º03'36" | 05º03'36" S | Maués (AM) | Oxisol | Projeto Radam |
| R16 | 57º10'48" | 05º35'24" S | Itaituba (PA) | Ultisol | Projeto Radam |
| R17 | 72º37'48" | 05º31'12" S | Guajará (AM) | Ultisol | Projeto Radam |
| R18 | 73º43'12" | 07º40'48" S | Mancio Lima (AC) | Alfisol | Projeto Radam |
| R19 | 72º45'00" | 08º11'24" S | Cruzeiro do Sul (AC) | Ultisol | Projeto Radam |
| R20 | 51º09'36" | 08º21'36" S | Cumaru do Norte (PA) | Ultisol | Projeto Radam |
| R21 | 72º48'00" | 08º21'36" S | Porto Walter (AC) | Alfisol | Projeto Radam |
| R22 | 51º29'24" | 08º34'12" S | Cumaru do Norte (PA) | Ultisol | Projeto Radam |
| R23 | 68º43'48" | 08º46'12" S | Boca do Acre (AM) | Alfisol | Projeto Radam |
| R24 | 51º31'12" | 08º46'48" S | Cumaru do Norte (PA) | Entisol | Projeto Radam |
| R25 | 52º04'12" | 09º02'24" S | São Felix do Xingu (PA) | Ultisol | Projeto Radam |
| R26 | 53º04'12" | 09º22'48" S | Altamira (PA) | Ultisol | Projeto Radam |
| R27 | 51º31'48" | 09º23'24" S | São Felix do Xingu (PA) | Entisol | Projeto Radam |
| R28 | 66º32'24" | 09º33'26" S | Lábrea (AM) | Oxisol | Projeto Radam |
| R29 | 52º21'36" | 09º38'24" S | São Felix do Xingu (PA) | Ultisol | Projeto Radam |
| R30 | 52º58'12" | 10º02'24" S | Peixoto de Azevedo (MT) | Entisol | Projeto Radam |
| R31 | 70º00'36" | 10º20'24" S | Sena Madureira (AC) | Inceptisol | Projeto Radam |
| R32 | 69º40'48" | 10º26'24" S | Sena Madureira (AC) | Alfisol | Projeto Radam |
| R33 | 64º59'24" | 10º26'24" S | Nova Mamoré (RO) | Oxisol | Projeto Radam |
| R34 | 64º09'00" | 10º34'48" S | Nova Mamoré (RO) | Entisol | Projeto Radam |
| R35 | 59º57'00" | 11º27'00" S | Juina (MT) | Entisol | Projeto Radam |
| R36 | 65º00'00" | 11º36'00" S | Guajará-Mirim (RO) | Entisol | Projeto Radam |
| R37 | 59º27'36" | 14º52'48" S | Pontes e Lacerda (MT) | Ultisol | Projeto Radam |
| R38 | 58º03'00" | 15º01'12" S | Salto do Ceu (MT) | Ultisol | Projeto Radam |
| R39 | 59º35'24" | 15º04'12" S | Vila Bela da Santa Trinidade (MT) | Oxisol | Projeto Radam |
